# Pathogenesis of Non-Arteritic Anterior Ischemic Optic Neuropathy Associated with COVID-19

**DOI:** 10.3390/ijms27062644

**Published:** 2026-03-13

**Authors:** Toshiyuki Oshitari

**Affiliations:** 1 Department of Ophthalmology and Visual Science, Chiba University Graduate School of Medicine, Inohana 1-8-1, Chuo-ku, Chiba 260-8670, Japan; tarii@aol.com; Tel.: +81-43-226-2124 or +81-43-224-4162; 2 Department of Ophthalmology, International University of Health and Welfare School of Medicine, 4-3 Kozunomori, Narita 286-8686, Japan

**Keywords:** non-arteritic ischemic neuropathy, COVID-19, angiotensin-converting enzyme 2, infection, endothelial cells, molecular pathology

## Abstract

Non-arteritic ischemic optic neuropathy (NAION) results from vascular insufficiency within the optic nerve head. The precise pathogenesis of NAION remains unclear; however, insufficient blood supply from the short posterior ciliary arteries and the choroidal circulation has been associated with its development. Although major risk factors include diabetes, hypertension, and hyperlipidemia, coronavirus disease 2019 (COVID-19) may also contribute to the development of NAION. This literature review presents our case of NAION associated with COVID-19 infection and summarizes previously reported cases of NAION following COVID-19 infection published in the English-language literature worldwide. Because direct infection of ocular tissues, including ocular vessels, via the angiotensin-converting enzyme 2 receptor is thought to contribute to the development of NAION, cases of NAION associated with COVID-19 vaccination were excluded from this review. Furthermore, we discuss the possible molecular mechanisms underlying the development of NAION after COVID-19 infection and highlight the potential risks of COVID-19 for clinical ophthalmologists.

## 1. Introduction

Non-arteritic anterior ischemic optic neuropathy (NAION) is one of the common optic neuropathies in adults and is thought to result from insufficient blood supply to the optic nerve head from the short posterior ciliary arteries and choroidal circulation [1,2]. Although the etiology of NAION is not fully understood, hypoperfusion or non-perfusion of the prelaminar region of the optic nerve head may be a potential risk factor for NAION. Known risk factors include cardiometabolic conditions such as diabetes mellitus, hypertension, and hyperlipidemia [3], a small and crowded optic disc [4,5], older age [6,7], nocturnal systemic arterial hypotension [8,9], obstructive sleep apnea syndrome [10,11], smoking [12], and certain medications such as semaglutide [13] or amiodarone [14].

Growing evidence indicates an association between coronavirus disease 2019 (COVID-19) infection with NAION. Since 2020, multiple case reports suggesting a possible association between severe acute respiratory syndrome coronavirus 2 (SARS-CoV-2)-infected COVID-19 and the development of NAION have been published [15,16,17,18,19,20,21,22,23,24,25]. In addition, posterior ischemic optic neuropathy following recurrent COVID-19 infection in patients with prior bilateral NAION has also been reported [26]. In most of the cases described above, inflammatory responses and hypercoagulability accompanied by vasculopathic risk factors are thought to be related to NAION; however, direct infection of SARS-CoV-2 in ocular tissues, including endothelial cells, may also partly contribute to the development of NAION. Nevertheless, the precise etiology of NAION associated with COVID-19 remains unclear.

In this article, we present our case of NAION associated with COVID-19 and summarize previously reported cases of NAION associated with COVID-19. Furthermore, we discuss possible molecular mechanisms underlying the development of NAION after direct SARS-CoV-2 infection. This review may be useful for clinical ophthalmologists to understand the possible mechanisms underlying the association between SARS-CoV-2 infection and the development of NAION.

## 2. Case Report

**Figure 1:**
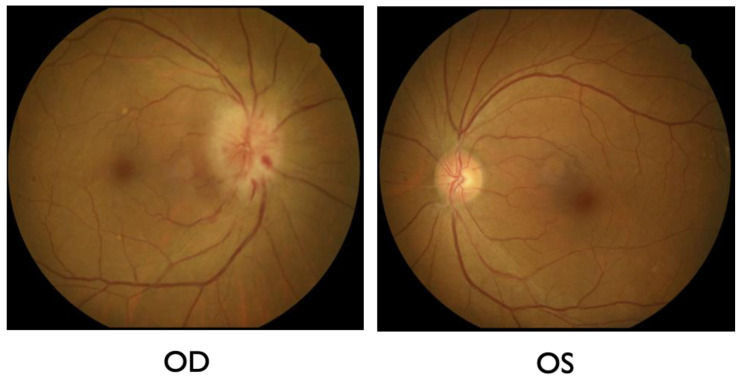
Fundus examination at the first visit. Right optic disc swelling with splinter hemorrhages at the inferior disc margin was found. Left optic disc was normal/healthy appearing.

A 66-year-old female was referred to the International University of Health and Welfare Narita Hospital because of a new subjective infratemporal visual field defect in the right eye 13 days prior to presentation. She had history of mild hypertension, which had been controlled with oral amlodipine besylate (2.5 mg) by her primary physician for over ten years. She had undergone uterine fibroid surgery 14 years prior. She had a COVID-19 infection one month before the onset of visual field defect in the right eye. She experienced flu-like symptoms and dizziness during the infection. At the first examination, her best-corrected visual acuities (BCVAs) were 0.7 (20/28.5) OD and 1.0 (20/20) OS. The critical fusion frequencies were 41 Hz (OD) and 44 Hz (OS). Colour vision testing with Panel D15 was normal for both eyes. A right relative afferent pupillary defect was present. On fundus examination, the patient had right optic disc edema with splinter hemorrhages at the inferior disc margin (Figure 1). The patient’s flu-like symptoms resolved at the first visit.

**Figure 2:**
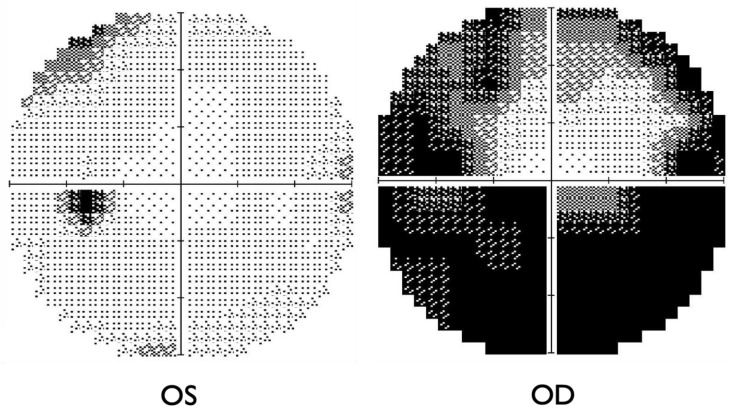
Humphry visual field test at the first visit. Right visual field showed a significant inferior altitudinal defect and a concentric contraction. Left visual field appeared grossly normal.

**Figure 3:**
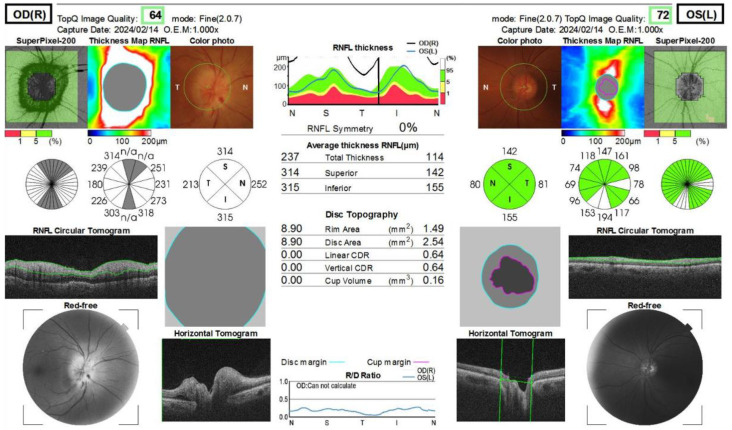
The OCT findings at the first visit. Right optic disc showed swelling and edema but left optic disc appeared normal. The green lines in the middle layer of the figure showed the measurement lines of the retinal nerve fiber layer thicknesses. The pink line showed the cup margin in the optic disc of left eye. Because of the disc swelling in the right eye, the cup margin of the right eye cannot be calculated. Therefore, the line graph in the bottom, only the line of disc margin (green) was displayed.

**Figure 4:**
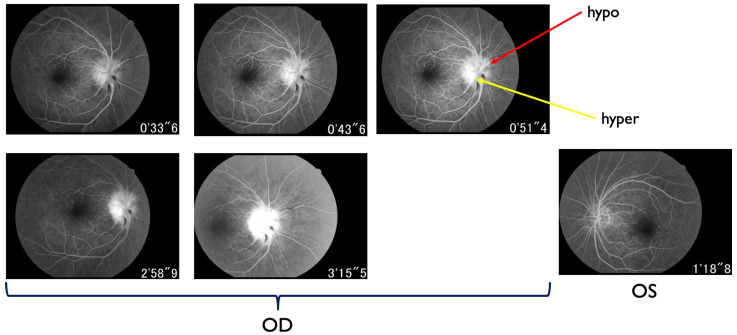
Fluorescence angiography findings at the first visit. Right optic disc had hypoperfusion areas at the early phase; however, at the late phase, the same area showed hyperfluorescence. These ischemic areas correspond to the visual field defect in Figure 2. There was also likely disc leakage OD as well. Left optic disc did not reveal any abnormal finding on fluorescence angiogram. Hypo; hypofluorescence. Hyper; hyperfluorescence.

**Figure 5:**
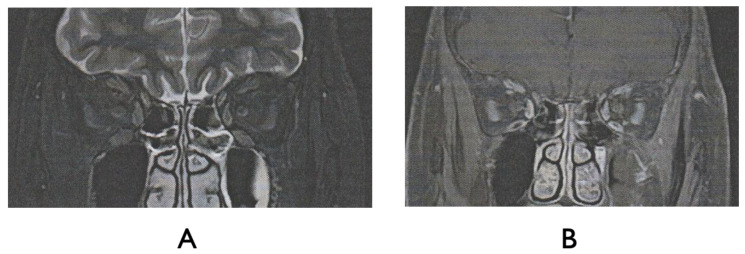
T2 short TI inversion recovery (STIR) (**A**) and Gadolinium-enhanced MRI (**B**) findings. No abnormal findings of the optic nerves were found on MRI; as such, there was low suspicion for optic neuritis as the cause of patient’s vision loss and right optic disc swelling.

The Humphrey visual field test showed significant inferior altitudinal defect and concentric contraction in the right eye (Figure 2), whereas the left visual field was grossly within normal limits. Optical coherence tomography (OCT) revealed swelling of the right optic disc, while the left optic disc appeared normal (Figure 3). Fluorescein angiography showed hypoperfusion of the right optic disc in the early phase; however, in the late phase, the right optic disc showed hyperfluorescence in the same area (Figure 4). These patterns were consistent with anterior optic disc ischemia, and the ischemic area corresponded to the visual field defect in the right eye (Figure 2). On review of symptoms, she denied headache or jaw claudication. T2 short TI inversion recovery (STIR) and Gadolinium-enhanced MRI revealed no abnormalities in either optic nerve (Figure 5). Blood tests showed no abnormal findings, including serum anti-aquaporin 4 antibody levels, erythrocyte sedimentation rate, or C-reactive protein levels. Myelin oligodendrocyte glycoprotein antibody was not routinely examined in our hospital. A relatively small cup-to-disc ratio (0.25) was found in the contralateral eye. Based on these findings, the final diagnosis was right NAION.

**Figure 6:**
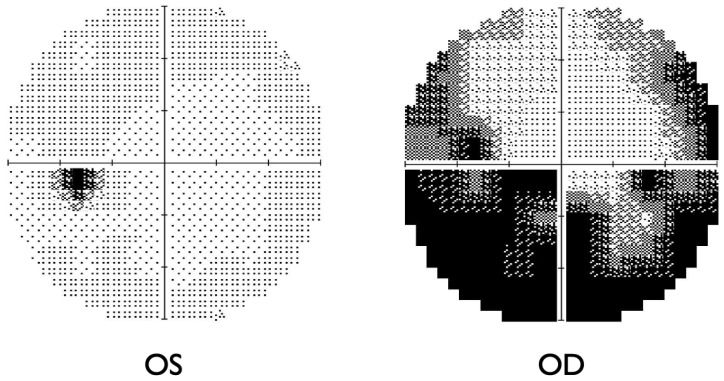
Humphry visual field test after 3 months of the treatment. The sensitivity of the centre of the right eye was slightly improved but the visual field defect remained grossly unchanged.

**Figure 7:**
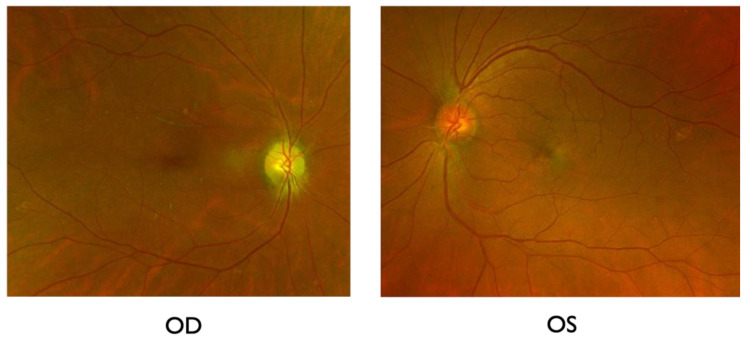
Colour fundus photographs after 3 months of the treatment. Although the optic disc edema in the right eye resolved, the disc became pale, consistent with prior ischemic damage. The cup-to-disc ratios (0.25) in both eyes were relatively small.

Vitamin B12, as a neuroprotectant and Kallidinogenase for improving blood circulation, was orally administered. To optimize the treatment of hypertension, the dose of amlodipine besilate (2.5 mg) was increased to 5.0 mg amlodipine besilate. Amlodipine besilate was taken in the morning. Her blood pressure decreased from 150/99 mmHg to 140/85 mmHg in the morning. She did not have episodes of obstructive sleep apnea. After 3 months of treatment, her BCVA improved from 0.7 (20/28.5) to 1.2 (20/16.7); however, the visual field defect in the right eye remained (Figure 6). Though the right disc edema improved, disc pallor was then apparent (Figure 7). Her visual acuity and field defects did not change for two years after treatment. No NAION developed in the left eye during the follow-up.

## 3. Discussion

The clinical findings and course of the patient in this case report were consistent with those of typical NAION. The patient did not have any findings of hypercoagulability but had relatively small cup-to-disc ratios (0.25) in both eyes. A small cup-to-disc ratio is known to be found with higher percentages in patients younger than 50 years with NAION [7]. The remaining questions were whether COVID-19 infection one month prior was related to the development of NAION and whether mild hypertension was, at least in part, associated with its onset.

To address the first question, we summarized previously reported cases of NAION following COVID-19 infection in Table 1. Our patient developed NAION one month after COVID-19 infection. Based on prior reports, the average time from COVID-19 infection to NAION onset was approximately 36.2 days (range, 0–210 days; median, 30 days) (Table 1) [15,16,17,18,19,20,21,22,23,24,25]. According to Shi et al.’s review of cases of neuro-ophthalmic sequelae following COVID-19 infection, optic neuritis was the most common condition after COVID-19 infection, with an average time to symptom onset of 32.8 days (range, 0–210 days) [27]. In another review regarding COVID-19-associated chorioretinal vasculopathy, Carletti et al. found that vascular occlusions, such as retinal vein or arterial occlusions, were the most frequently reported conditions, with an average onset of approximately 30 days after infection [28]. Naber et al. reported the case of NAION seven months after COVID-19 infection [21]. In their case, the patient had no history of vascular risk factors (such as hypertension or diabetes) and did not receive COVID vaccination preceding diagnosis of NAION. The patient was a non-smoker and exhibited a normal electrocardiogram, pulmonary function test results, and brain/orbit MRI [21]. They finally concluded that subtle endothelial injury induced by COVID-19 infection remains present beyond the acute phase [21]. Taken together, the one-month delay between COVID-19 infection and NAION onset in our case appears to coincide with the typical time frame reported after infection.

Second, hypertension is a common risk factor for NAION and it increases the odds of developing NAION by approximately 1.5-fold [3,29]. In general, hypertension leads to retinal arteriolar narrowing, followed by impairment of optic nerve circulation [30]; therefore, hypertension may accelerate the development of NAION after COVID-19 infection. Savastano et al. reported that radial peripapillary capillary plexus (RPCP) perfusion density was lower in post-SARS-CoV-2 patients (n = 80) than in controls (n = 30), and that, within the post-COVID-19 group, patients with systemic arterial hypertension (n = 19) had a lower RPCP flow index [31]. Thus, even mild hypertension may exacerbate circulation impairment around the prelaminar region of the optic nerve head and may have contributed to the development of NAION after COVID-19 infection in our patient.

Currently, the pathogenesis of NAION following COVID-19 infection is still not fully understood; however, immune-mediated mechanisms, hypercoagulability, endothelial cell damage, and underlying vasculopathic risk factors may be involved [15,16,17,18,19,20,21,22,23,24,25].

Although this remains a personal opinion, direct infection of SARS-CoV-2 in ocular tissues, including endothelial cells surrounding the optic nerve head, may also be related to the onset of NAION.

There are several reasons for the possible association between direct SARS-CoV-2 infection with ocular tissues, including endothelial cells surrounding the optic nerve head, and the development of NAION.

**Figure 8:**
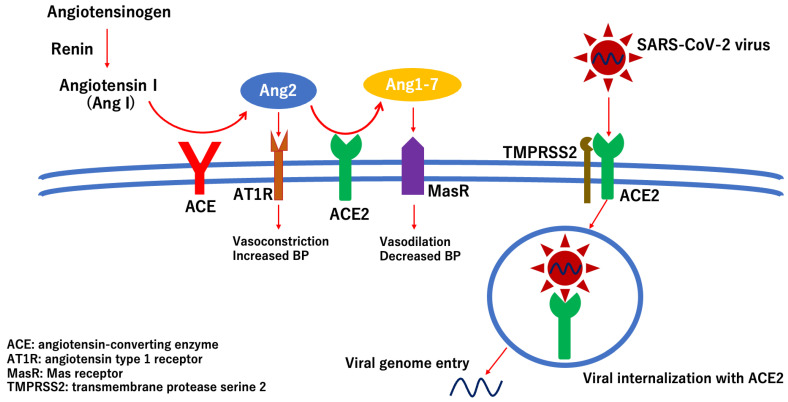
Scheme of the mechanism of SARS-CoV infection in host cells. Angiotensin-converting enzyme 2 (ACE2) catalyzes the conversion of angiotensin II to angiotensin 1–7, resulting in vasodilation and decreased blood pressure (BP) via the Mas receptor. SARS-CoV-2 enters host cells via the ACE2 transmembrane receptor. Transmembrane protease serine 2 (TMPRSS2) supports viral attachment to the host cell surface by activating viral fusion proteins. After binding to the ACE2 receptor, the virus–receptor complex is translocated to the endosome of the infected cell.

Angiotensin-converting enzyme 2 (ACE2) is a transmembrane receptor involved in the renin–angiotensin system that catalyzes the conversion of angiotensin II to angiotensin 1–7 followed by a decrease in blood pressure [32]. SARS-CoV-2 enters host cells via the ACE2 receptor (Figure 8) [33,34,35]. Transmembrane protease serine 2 (TMPRSS2) facilitates viral attachment to the surface of the target cell by activating viral fusion proteins (Figure 8) [36].

Hill et al. analyzed ACE2 expression in ocular tissues of aged humans and found ACE2 protein expression in the corneal endothelial cells, trabecular meshwork cells, non-pigmented ciliary epithelial cells, ocular choroid fibroblasts, whole retina, and optic nerve [37]. Additionally, Zhou et al. detected ACE2 expression in primary human retinal endothelial cells and primary human retinal pericytes, and TMPRSS2 expression in retinal neuronal cells, vascular and perivascular cells, and Müller cells [38]. These studies demonstrate that ocular tissues, including the optic nerve, can be directly infected with SARS-CoV-2. In fact, a recent study indicated that human-induced pluripotent stem cell-derived retinal organoids expressed the ACE2 receptor and TMPRSS2, and that the SARS-CoV-2 pseudovirus could infect the retinal organoid [39]. Another study suggested that SARS-CoV-2 can infect and replicate in the photoreceptors and retinal ganglion cells of human retinal organoids prepared using human-induced pluripotent stem cells [40]. This study highlighted the possible long-term effects of SARS-CoV-2 infection on ocular tissues. Furthermore, Sen et al. performed in situ hybridization to determine the cellular localization of the SARS-CoV-2 spike gene RNA in postmortem eyes obtained from 25 patients with COVID-19. Their results indicated that SARS-CoV-2 RNA was localized in the inner and outer retinal layers, ganglion cell layers, corneal epithelia, scleral fibroblasts, and oligodendrocytes of the optic nerve [41]. Furthermore, the long-term effect of SARS-CoV-2 infection can exist in infected cells [40], and the late development of NAION after COVID-19 has been reported [21]. In fact, beyond NAION, several patients with late development of optic neuritis after COVID-19 have also been reported: 60 days following infection [42], 210 days after infection [43], and 180 days following infection [44]. Taken together, the effect of direct SARS-CoV-2 infection on the ocular tissues cannot be ignored when elucidating the pathogenesis of NAION associated with COVID-19.

**Figure 9:**
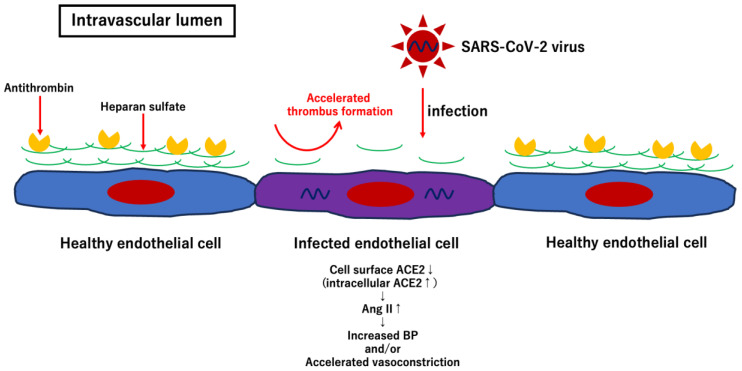
Endothelial cell dysfunction and mechanisms of hypercoagulation after direct infection of SARS-CoV-2 virus into endothelial cells. SARS-CoV virus and damage-associated molecular patterns stimulate monocytes which result in releasing inflammatory cytokines and activating coagulation. In addition, once SARS-CoV-2 virus infects endothelial cells, glycocalyx on the cell surface composed of heparan sulfate, hyaluronic acid, proteoglycan and antithrombin is degraded, followed by accelerating thrombus formation at the local area.

In addition to affecting various ocular tissues, SARS-CoV-2 can also directly infect endothelial cells as they express the ACE2 receptor and TMPRSS2 [38]. Once SARS-CoV-2 infects endothelial cells, the glycocalyx on the endothelial cell surface, which is composed of glycosaminoglycans, such as hyaluronic acid or heparan sulfate, proteoglycan, and antithrombin, is degraded, resulting in accelerated thrombus formation in the local area [45,46,47]. Furthermore, after infection with SARS-CoV-2, ACE2 expression on the cell surface is downregulated, followed by an increase in angiotensin II levels [48]. Consequently, the effect of angiotensin II via the angiotensin type I receptor is enhanced by increasing blood pressure and accelerating vasoconstriction (Figure 8 and Figure 9) [49,50]. At least five patients with NAION associated with COVID-19 have no known vascular risk factors for developing NAION (Table 1). Even without risk factors, once SARS-CoV-2 infects the endothelial cells of the vessels surrounding the optic nerve head, hypercoagulation and vascular occlusion can occur in the infected area, resulting in the development of NAION. In summary, I propose a potential scheme to depict the pathologic effects of direct infection of SARS-CoV-2 virus into endothelial cells, thereby leading to the development of NAION (Figure 9).

As mentioned above, direct infection with SARS-CoV-2 induces endotheliitis by exposing the thrombogenic basement membrane, followed by activation of thrombus formation (Figure 9) [51]. In addition, SARS-CoV-2 spike proteins activate complement-associated microvascular injury and facilitate thrombus formation [52]. Because the direct infection of ocular tissues with SARS-CoV can be associated with the development of NAION and other ocular diseases such as uveitis, retinal vein/artery occlusion, or optic neuritis, the systemic severity of COVID-19 does not seem to be related to the development and severity of ocular manifestations. One possible reason is that ocular tissues can serve as reservoirs for viral replication after infection [40]. Although the precise pathogenesis of NAION associated with COVID-19 remains unclear, direct infection of SARS-CoV-2 virus into ocular tissues, including the endothelial cells of vessels surrounding the optic nerve head, may be associated with the development of NAION after COVID-19. Further studies and accumulation of similar cases are required to elucidate the pathogenesis of NAION in COVID-19.

## 4. Future Perspectives for Therapeutic Options

Therapeutic options and their effectiveness for NAION are controversial, and from our personal point of view, there have been no established standard treatment options for NAION at this point. One reason for the lack of a standard therapy for NAION is that approximately 30% of patients with NAION without treatment spontaneously recovered [53,54]; as such, it is difficult to precisely evaluate the effect of NAION treatment. In our case, oral vitamin B12 and Kallidinogenase were administered because of their safety profiles, and the patient’s visual acuity and visual field defects improved slightly. However, this improvement appeared to be spontaneous and natural.

Aspirin and corticosteroids are frequently used to treat NAION worldwide [55]. However, some large retrospective case–controlled studies have indicated that aspirin shows no treatment effect in patients with NAION and does not reduce the risk of NAION in the contralateral eye [56,57]. Similarly, a recent randomized clinical trial and meta-analysis suggested that corticosteroids did not significantly improve visual outcomes in patients with NAION [58,59]. In addition, corticosteroids increase the risk of cardiovascular complications, probably due to the procoagulant state [60], and thus may increase the risk of NAION development in the contralateral eye. In fact, among the 12 cases, including ours, 4 developed NIAON in both eyes, and 2 were treated with oral steroid therapy (Table 1). Usually, the risk of developing NAION in the contralateral eye is approximately 12–15% [61]. Therefore, we did not treat the patient with oral steroid. To date, no beneficial treatment has been identified to prevent NAION in the contralateral eye.

In search of a potential acute treatment option or preventive measure for NAION, several neuroprotective therapies have been used; positive results have been partially obtained in small studies for therapies such as intravitreal erythropoietin injection [62], oral citicoline administration [63], or subcutaneous injection of gum mastic extract RPh201 [64]. However, further randomized clinical trials are required to examine the neuroprotective effects of these drugs against NAION.

## 5. Conclusions

There are still patients being infected with COVID-19 in Japan and worldwide, thereby potentially increasing the risk of NAION development in these patients. Although the etiology of NAION after COVID-19 remains unknown, direct SARS-CoV-2 infection in the endothelial cells of the vessels surrounding the optic nerve head may be associated with the development of NAION. Clinical ophthalmologists should be aware that COVID-19 is a risk factor for NAION development.

## Figures and Tables

**Table 1:** Summary of previous cases of NAION possibly associated with COVID-19 infection. Please notice that the average times from the COVID-19 infection to the onset of NAION were approximately 36.2 days (range 0–210 days). Cases presented in references [15,24] developed NAION in both eyes after steroid therapies. At least five cases had no risk factors for NAION. In cases of NAION developed in both eyes, even with time lags, the term “Bilateral” is stated in the column of clinical findings. DM, diabetes mellitus; VA, visual acuity; CF, counting finger; GCLIP, ganglion cell-inner plexiform layer; BP, blood pressure; HM, hand motion; RNFL, retinal nerve fibre layer; ARDS, acute respiratory distress syndrome; VF, visual field; HT, hypertension; LP, light perception.

	Patient Data	Duration from Infection to Onset	Clinical Findings	Treatment and Prognosis	Mechanisms
Rho et al. 2020 [20]	45 y.o. male, DM(+), borderline hyperlipidemia	1 month	VA = 20/30 (OD), inferior altitudinal defect (OS)	Optimize vasculopathic risk factors by primary care physicians, no description of prognosis	Vasculopathic risks + hypercoagulation and low-oxygen state by COVID-19 infection
Yüksel et al. 2021 [19]	72 y.o. male, controlled DM(+)	13 days	VA = 0.3 (OD), inferior altitudinal defect (OD)	Aspirin was already prescribed. No additional treatment was performed. Visual acuity was decreased to CF. Progressive GCLIP atrophy	Additional risk of COVID-19 infection to vasculopathic risks
Moschetta et al. 2021 [18]	64 y.o. male, no risk factors but BP = 150/90 mmHg after hospital discharge, low hydroxy vitamin D	5 weeks	VA = 20/20 (OD). inferior altitudinal defect (OD)	Vit D supplementation, valsartan with aspirin for secondary cardiovascular protection, progress of visual field defect, VD = 20/20	COVID-19-associated endotheliopathy
Golabchi et al. 2021 [17]	52 y.o. male, no risk factors	2 weeks	VA = HM (OD), generalized visual field defect with central and nasal scotoma, diffuse RNFL thinning	No treatment	Severe inflammatory response and hypercoagulability
Clarke et al. 2021 [16]	55 y.o. male, prone position	0 day	VAs = CF (OD), and 3/30 (OS), constricted visual field defect (OD), depressed inferonasal vision (OS) (Bilateral)	No treatment	Prone position for ARDS
Sitaula et al. 2022 [25]	60 y.o. female, no risk factors	6 days	VA = 20/200 (OS), inferior altitudinal defect (OS)	Aspirin, VA was improved to 20/32 but VF defect was remained	Inflammation and endothelial damage
Sanoria et al. 2022 [15]	45 y.o. male, DM(+), HT(+), well controlled	1 month (first developed in the right/after 2 weeks, left eye had NAION)	VA = 6/6 (OD), and 6/24 (OS), inferior field defect (OD), concentric constriction (OS) (Bilateral)	Oral steroid, no effect	Immuno-mediated pathogenesis post-viral infection
Kiyat et al. 2023 [24]	50 y.o. no risk factors	1 month (first developed in the left/after 3 weeks, right eye had NAION)	VAs = 8/10 (OD), and 3/10 (OS), blind spot enlargement (OD), inferior altitudinal defect (OS) (Bilateral)	Oral steroid + antiplatelet agent, no effect (OD), steroid pulse therapy + antiplatelet agent, no effect (OS)	Immuno-mediated mechanisms, steroid may affect the eye and lead to NAION development
Vosoughi et al. 2023 [23]	60 y.o. female, DM(+), HT(+), anemia	1 month	VAs = 20/20 (OD), and CF (OS), superior altitudinal defect (OD), dense complete visual field defect (OS) (Bilateral)	Optimize hematological parameters (prevent anemia, control HT and DM), visual function remained unchanged	Severe epistaxis and anemia (hemorrhagic-induced NAION)
Romozzi et al. 2023 [22]	61 y.o. no risk factors	0 day, vaccine after 6 months the onset worsened NAION	VAs = 20/20 (OD), and 20/25 (OS), after vaccine administration, VA = LP (OS), concentric reduction (OS), after vaccine administration, total loss of visual field (OS)	Oral prednisone, no effect	Microangiopathic/thrombotic events
Naber et al. 2024 [21]	post-menopausal, female, no risk factors	7 months	VA = 20/20 (OD), inferotemporal visual field loss (OD) (not typical)	Steroid pulse therapy, VF was slightly improved	Persistent endothelial damage

## Data Availability

The original contributions of this study are presented in this article. Further inquiries can be directed to the corresponding author.
